# Dataset on cardiac structural and functional parameters in TMAO-challenged mouse

**DOI:** 10.1016/j.dib.2024.110465

**Published:** 2024-04-24

**Authors:** Yingyu Wang, Chenchen Qin, Shiyi Tian, Yuqin Meng, Yige Chen, Sunjing Fu, Mengting Xu, Bing Wang, Yuan Li, Qin Ouyang, Hao Ling, Mingming Liu

**Affiliations:** a Institute of Microcirculation, Chinese Academy of Medical Sciences & Peking Union Medical College, Beijing 100005, China; b International Center of Microvascular Medicine, Chinese Academy of Medical Sciences, Beijing 100005, China; c School of Nursing, Chinese Academy of Medical Sciences & Peking Union Medical College, Beijing 100144, China; d Department of Pathology, Wangjing Hospital, China Academy of Chinese Medical Sciences, Beijing 100102, China; e Department of Radiology, The Affiliated Changsha Central Hospital, Hengyang Medical School, University of South China, Changsha 410000, Hunan, China; f Diabetes Research Center, Chinese Academy of Medical Sciences, Beijing 100005, China

**Keywords:** Trimethylamine-N-oxide, Echocardiograph, Cardiovascular diseases, Heart rate, Ejection fraction

## Abstract

Trimethylamine-N-oxide (TMAO) is a gut-derived metabolite formed from dietary choline and l-carnitine, known to impede cholesterol metabolism and is implicated in the pathogenesis of thrombosis and atherosclerosis, contributing to the etiology of cardiovascular diseases. We present a dataset derived from an experimental study designed to elucidate the cardiotoxic effects of TMAO. This dataset encompasses echocardiographic assessments from two cohorts of mice: one subjected to a 6-week regimen of 20 mg/kg/day TMAO injections (*n* = 16) and a control group (*n* = 18). Each subject's echocardiographic dataset comprises six high-resolution TIFF images, capturing both B-type and M-mode views in standard echocardiographic planes, along with two additional M-mode images enriched with analysed cardiac functional data. Complementing these images, a CSV-formatted report details critical cardiac parameters, including heart rate, ejection fraction, and fractional shortening, among others. In a novel approach to enhance data integrity and permit tailored analyses, we provide the original output files from the echocardiography apparatus, which researchers can reprocess using dedicated analysis software. This dataset is anticipated to be instrumental in advancing our understanding of the mechanistic links between TMAO exposure and cardiac dysfunction.

Specifications Table

**Table:** 

Subject	Cardiology and Cardiovascular Medicine, Endocrinology and metabolism
Specific subject area	Trimethylamine-N-oxide challenged cardiovascular disease
Data format	Raw: BIMG, MXML, BAK, PIMG, PNG, VXML and PVD Analysed: TIF and CSV
Type of data	Figures and Tables
Data collection	Cardiac ultrasonography was performed utilizing the Fujifilm Visual Sonics 3100 system to obtain echocardiographic representations of murine cardiac models using both M-mode and B-mode imaging techniques from parasternal long-axis and apical views. Image acquisition yielded raw files in the TIFF format, each with a resolution of 1920 × 992 pixels. Data collection transpired on the 28th of November and the 6th of December 2023, within the confines of the Institute of Laboratory Animal Science. Subsequent to collection, these primary images underwent meticulous manual inspection and quantification employing the Vevo LAB software, version 5.7.0. The analytical process resulted in generation of detailed images that delineated cardiac structural parameters, accompanied by individual Microsoft Excel spreadsheets summarizing the data for each murine subject.
Data source location	Institution: Institute of Microcirculation, Chinese Academy of Medical Sciences & Peking Union Medical College, International Center of Microvascular Medicine, Chinese Academy of Medical Sciences City/Town/Region/Country: Beijing, Dong Cheng District, China
Data accessibility	Repository name: Figshare Data identification number: 10.6084/m9.figshare.25351405 Direct URL to data: https://figshare.com/s/1a39df0713c1f8497769

## 1 Value of the Data

• The comprehensive dataset offers high-resolution B-type and M-type echocardiographic images alongside detailed functional parameters from TMAO-challenged mouse models, providing a robust platform for investigating the mechanistic links between gut-derived metabolites and cardiovascular disease [1]. This facilitates a deeper understanding of TMAO's role in cardiac pathology and its potential as a biomarker for cardiovascular risk [2,3].
• By including raw output files from echocardiographic assessments, the dataset allows for reproducible and refined analyses with the potential to reduce human error. This feature ensures that researchers can independently verify findings and explore novel analytical approaches, enhancing the reliability and scientific rigor of cardiovascular research.
• The dataset serves as a resource for the development of targeted interventions by elucidating the cardiotoxic effects of TMAO. It aids in the identification of therapeutic targets [4], contributing to the advancement of precision medicine in cardiovascular disease prevention and management [5].

## 2 Background

Trimethylamine N-oxide (TMAO) is emerging as a biomolecule of considerable interest within cardiovascular pathophysiology. A growing body of evidence implicates elevated TMAO levels in the etiology of various cardiovascular diseases (CVDs), rendering it a novel target of interest within the biomedical and clinical research communities. Despite this burgeoning recognition, the precise impact of TMAO on cardiac morphology and function remains incompletely elucidated. To this end, we designed a controlled experimental study to delineate the effects of TMAO on the myocardium. Utilizing a 6-week TMAO intervention protocol in murine models, we systematically assessed cardiac parameters in comparison with a matched control cohort via echocardiography.

## 3 Data Description

The dataset underpinning this investigation is stratified into two distinct subsets: the primary analyzed dataset, conducive to the execution of explicit differential assessments, and the auxiliary corpus of unprocessed raw data, emanating directly from the echocardiographic software, which affords the flexibility for indefinite re-evaluation of cardiac metrics. The curated analyzed dataset comprises a compendium of echocardiographic images and corresponding tabular records for a cohort of 16 murine models subjected to a 6-week dietary regimen enriched with TMAO, in juxtaposition with a control contingent of 18 specimens. Each subject's data portfolio encompasses a sextet of images, inclusive of both M-mode and B-mode echocardiographic projections, captured from the parasternal long-axis and apical four-chamber orientations, alongside a duo of supplementary M-mode representations with embedded cardiac functional parameters.

The quantitative structural parameters delineated within the accompanying Excel spreadsheets are derived from a rigorous analysis of M-mode echocardiographic sequences. The accompanying CSV file in each folder is methodically structured to encapsulate echocardiographic measurements from the involved experiments. The file initiates with a descriptive header that clarifies the file's purpose and the date of report generation. Subsequent sections delineate the utilized hardware and software, specifically noting the employment of Vevo LAB, version 5.7.0, thereby ensuring reproducibility and consistency in data acquisition. Furthermore, the file documents metadata pertinent to the study, including the study name, date, and administrator information, ensuring the traceability and accountability of the data collection process. Details concerning the series further elucidate the timing and personnel involved in data acquisition, enhancing the contextual understanding of the dataset. The core section of the document systematically lists detailed echocardiographic measurements for various cardiac regions, including heart rate, volumes, and dimensions. These are presented in a structured tabular format, specifying parameters, units, and corresponding values, thereby furnishing a robust dataset conducive to comprehensive cardiovascular research analysis.

**Fig. 1:**
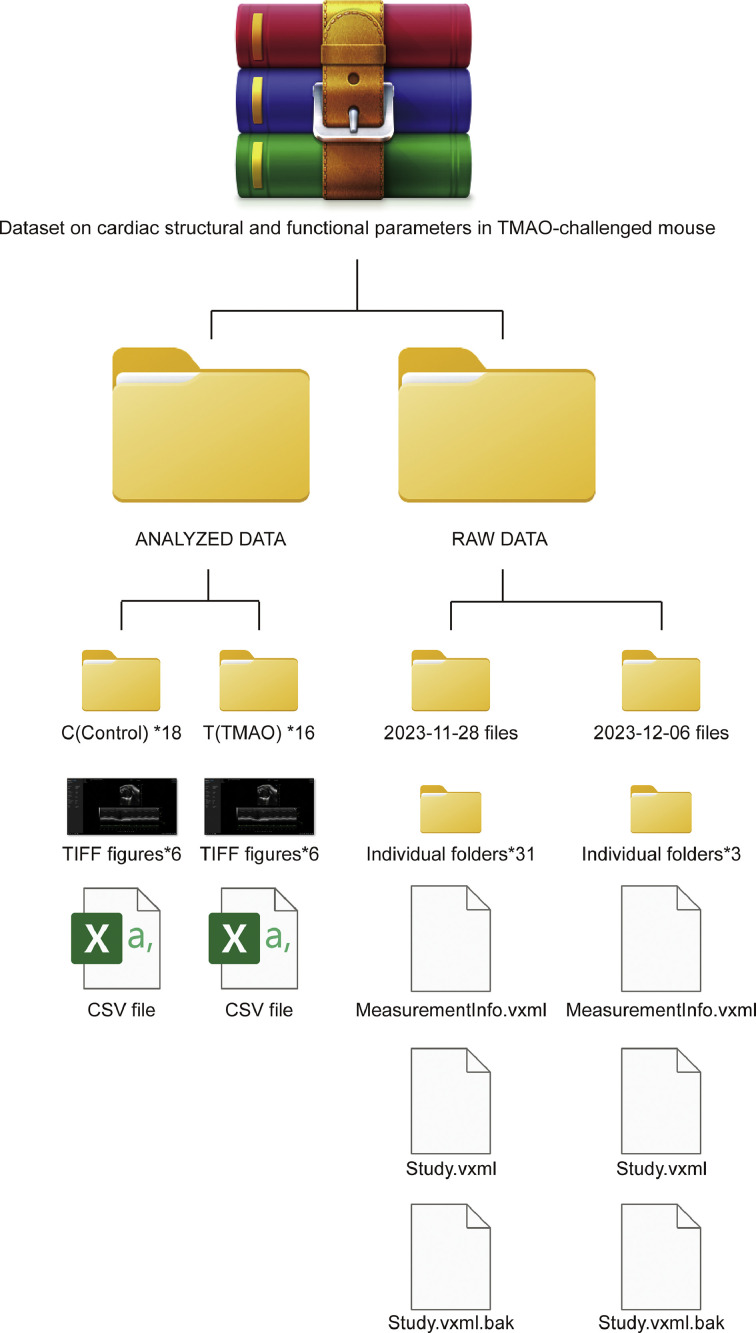
Organizational chart of file directories for TMAO-challenged mouse cardiac data. This architecture illustrates the hierarchical organization of the dataset, which is bifurcated into two primary directories: 'ANALYZED DATA' and 'RAW DATA.' The 'ANALYZED DATA' directory is further subdivided by the experimental groups, control (C) and TMAO-treated (T), with 18 and 16 replicate folders respectively. Each of these replicate folders contains six TIFF images of cardiac visualization and a corresponding CSV file with quantified data. The 'RAW DATA' directory is organized chronologically, as indicated by the date-specific folders, reflecting the temporal sequence of data acquisition. Within each date-specific folder, individual subject folders are allocated, each encompassing raw image files and XML files detailing the measurement parameters and study metadata. The XML backups are denoted with a '.bak' extension, ensuring data integrity and reproducibility of the study.

The acquisition of all echocardiographic images was conducted by specialists in the field, ensuring adherence to a uniform resolution of 96 dots per inch (dpi) and dimensions of 1920 pixels in width by 992 pixels in height. This standardization provides a consistent basis for subsequent image analyses. The raw data directory houses a curated echocardiographic dataset, documenting the cardiac structural and functional parameters of these subject mice. At the apex of the directory's hierarchy, the structure bifurcates into two temporal subdirectories, each corresponding to the dates of data acquisition. These date-stamped subdirectories are further divided into individual folders, organizing the data to facilitate easy access and analysis. Within each subdirectory, files critical for the reconstruction and analysis of the echocardiographic data are systematically organized. The “MeasurementInfo.vxml” files, formatted in XML, contain essential metadata about the echocardiographic measurements, including the parameters monitored, units of measurement, and the metadata schema employed. This ensures the consistency and interpretability of the data across different analytical platforms. The “Study.vxml” files comprise the raw data outputs from the echocardiographic studies, formatted in a manner that is readily ingested by specialized analysis software. Additionally, the inclusion of backup files, denoted by the “.bak” extension, serves as a safeguard against potential data loss or corruption. Besides, Table 1 presents a detailed overview of the dataset's attributes, while Fig. 1 illustrates the organizational structure of the data storage, providing a clear visual guide to the dataset's architecture.

**Table 1:** Descriptive summary of TMAO-challenged mice and echo-derived cardiac measurements.

No.	Particulars	Description
1	Mouse stains	C57BL/6N
2	Group setting	TMAO (*n* = 16) vs Control (*n* = 18)
3	Drug intervention	20 mg/kg/day TMAO vs 20 mg/kg/day boiled water for continuous 42 days (6 weeks)
4	Ultrasound cardiograph Acquisition	Measured by Fujifilm Visual Sonics 3100 and analysed with Vevo LAB 5.7.0.

**Fig. 2:**
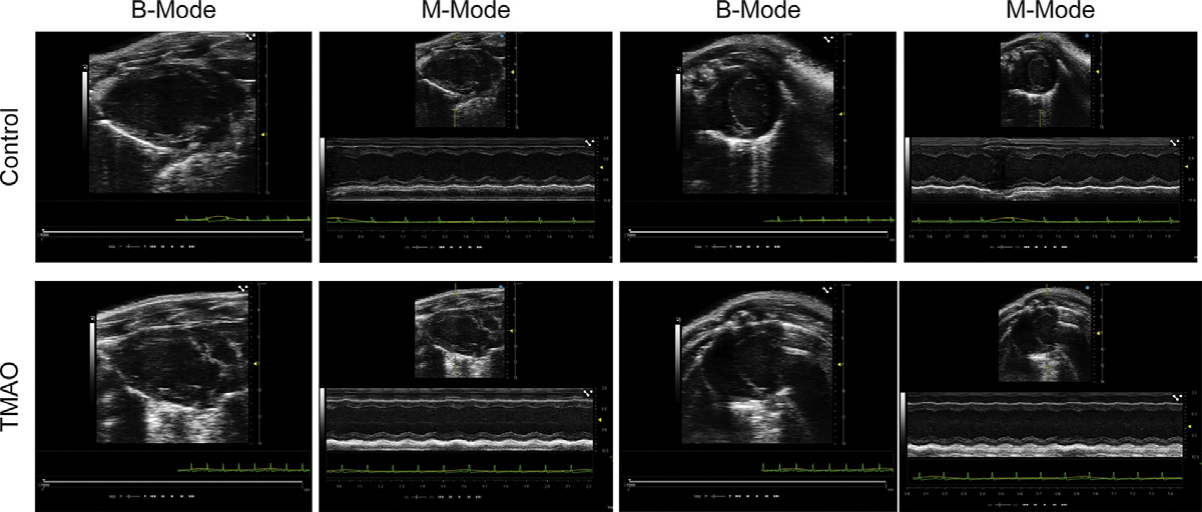
Echocardiographic assessment of murine heart structure and function in control and TMAO-challenged groups. The upper panel displays B-Mode and M-Mode echocardiograms from control mice, while the lower panel presents the same from TMAO-challenged mice. B-Mode images provide a two-dimensional view of the cardiac anatomy, allowing for visualization of chamber structure and wall thickness. M-Mode images capture the longitudinal motion of the heart, providing information on the dynamic changes in cardiac dimensions during the cycle. Accompanying each echocardiogram is an electrocardiogram trace (green line) to correlate electrical activity with mechanical function.

An example of echocardiographs is shown in Fig. 2. The image displays echocardiographic images of murine hearts, comparing control mice to those challenged with TMAO. Each row represents one group, with the control group on top and the TMAO group below. For each group, there are two sets of images: B-Mode (left) and M-Mode (right) echocardiograms. B-Mode, or 2D mode, provides a two-dimensional image of the heart, allowing for the visualization of heart structures. M-Mode, or motion mode, captures the movement and dimensions of the heart structures over time, which is displayed as a single-line trace that shows the motion of the heart during the cardiac cycle. In the control group, the B-Mode images show clear and defined cardiac chambers and walls, while the M-Mode images show consistent and rhythmic contractions of the heart muscle. While in the TMAO group, the B-Mode images may show variations in heart structure compared to the control, which could indicate changes due to TMAO treatment, such as alterations in chamber size or wall thickness. Similarly, the M-Mode images might show differences in the motion pattern of the heart, which could suggest alterations in cardiac function. The green lines at the bottom of each image represent the electrocardiogram, which records the electrical activity of the heart and is synchronized with the echocardiographic images. This type of comparison can be used to assess the impact of TMAO on cardiac structure and function in a research setting. These comparative images are critical for evaluating the cardiotoxic effects of TMAO and elucidating potential structural and functional alterations in the heart muscle.

## 4 Experimental Design, Materials and Methods

### 4.1 Mouse strain and pharmacological intervention

We employed male C57BL/6 N mice aged 8 weeks to establish a model for investigating the cardiovascular implications of TMAO. The study design included a TMAO intervention group and a matched control set. The intervention entailed administering a daily intraperitoneal injection of TMAO at a dosage of 20 mg/kg for a duration of 42 consecutive days [6]. The control group received an equivalent volume of sterile, pyrogen-free water to ensure the elimination of any potential contaminants. All mice were maintained under stringent laboratory conditions with a consistent light-dark cycle, temperature, and humidity control. The administration of injections was meticulously performed by a single trained technician to ensure consistency in dosing and handling stress.

### 4.2 Echocardiographic data acquisition

Prior to detailed echocardiographic imaging, we prepared the mice by applying a depilatory agent to the anterior chest region to remove fur and minimize image artifacts without causing dermal abrasion. Echocardiography was conducted under isoflurane anesthesia to achieve muscle relaxation and mitigate motion artifacts, ensuring the acquisition of high-fidelity cardiac images. The echocardiographic study was carried out by echocardiographers with expertise in murine models. Comprehensive measurements were taken, including but not limited to heart rate, left ventricular systolic and diastolic diameters and volumes, ejection fraction, and fractional shortening. These parameters were recorded to facilitate subsequent comparative analyses between the TMAO-challenged group and the controls.

The echocardiographic assessment was standardized to ensure reproducibility and accuracy, with a focus on capturing high-resolution B-mode and M-mode images that provide a detailed representation of cardiac function and morphology. The resulting echocardiographic data, indicative of TMAO's impact on cardiovascular health, has been curated with the intent of offering a robust foundation for future research endeavors seeking to delineate the cardiac ramifications of dietary metabolites.

## Limitations

The dataset underpinning the investigation of TMAO's effects on cardiac health, while robust in its ultrasound cardiograph presentation, is subject to several limitations. The usage of a 6-week TMAO injection regimen in mice may not accurately emulate the chronic exposure seen in human dietary patterns, raising concerns about the direct applicability of the results to human cardiovascular disease. Moreover, interspecies differences necessitate cautious extrapolation of murine data to human physiology. Though the provision of raw output files for re-analysis is a strength, it also presupposes the availability of specific analytical software and expertise, which may not be universally accessible. While the dataset offers valuable insights into the TMAO-cardiac disease nexus, it should be considered within the larger mosaic of cardiovascular research, acknowledging the complexity of disease mechanisms beyond the scope of a single metabolite.

## Ethics Statement

The present investigation was conducted with the endorsement of the Institutional Animal Care and Use Committee (IACUC) of the Chinese Academy of Medical Sciences (CAMS) under the approval number CAMS-IM-IACUC-2023-AE-0928. Our methodology strictly adhered to the established guidelines for the Care and Use of Laboratory Animals. These male murine subjects were housed in individual enclosures, with environmental conditions meticulously regulated at a constant temperature of 22 °C and a relative humidity maintained within a 55–70 % range. The housing facility ensured a rigorous adherence to a photoperiod regimen consisting of 12 h of light followed by 12 h of darkness. Throughout the duration of the study, the mice had ad libitum access to standard rodent chow and water, ensuring their physiological and metabolic needs were consistently met.

## CRediT authorship contribution statement

**Yingyu Wang:** Project administration, Software, Writing – original draft. **Chenchen Qin:** Validation, Writing – review & editing. **Shiyi Tian:** Validation, Writing – review & editing. **Yuqin Meng:** Validation, Writing – review & editing. **Yige Chen:** Validation, Writing – review & editing. **Sunjing Fu:** Validation, Writing – review & editing. **Mengting Xu:** Validation, Writing – review & editing. **Bing Wang:** Validation, Writing – review & editing. **Yuan Li:** Validation, Writing – review & editing. **Qin Ouyang:** Validation, Writing – review & editing. **Hao Ling:** Validation, Writing – review & editing. **Mingming Liu:** Supervision, Validation, Writing – review & editing.

## Data Availability

Dataset of contrast of cardiac structural and functional parameters in TMAO mice models (Original data) (figshare).
